# Beyond Anti-VEGF: Toward Integrated Precision Care in Diabetic Retinal Disease

**DOI:** 10.3390/pharmaceutics18091178

**Published:** 2026-09-17

**Authors:** Jesus H. Gonzalez-Cortes, Jesus E. Gonzalez-Cantu, Alper Bilgic, Francesc March de Ribot, Aditya Sudhalkar, Laurent Kodjikian, David Pelayes, Thibaud Mathis, Jesus Mohamed-Hamsho

**Affiliations:** 1 Department of Ophthalmology, University Hospital, Faculty of Medicine, Universidad Autónoma de Nuevo León (UANL), Monterrey 64460, Nuevo León, Mexico; emiliano_gzz@hotmail.com (J.E.G.-C.); jesus.mohamedhms@uanl.edu.mx (J.M.-H.); 2 OSG Augen-Tagesklinik Wittenberge, 19322 Wittenberge, Germany; drbilgicalper@yahoo.com; 3 Department of Medicine (Ophthalmology), University of Otago, Dunedin 9016, New Zealand; marchfrancesc@gmail.com; 4 Department of Ophthalmology, University of Girona, 17004 Girona, Spain; 5 MS Sudhalkar Medical Research Foundation, Baroda 390001, India; adityasudhalkar@yahoo.com; 6 Department of Ophthalmology, Croix-Rousse University Hospital, Hospices Civils de Lyon, Université Claude Bernard Lyon 1, 69004 Lyon, France; laurent.kodjikian@chu-lyon.fr (L.K.); mathisthibaud@hotmail.fr (T.M.); 7 UMR5510 MATEIS, CNRS, INSA Lyon, Université Claude Bernard Lyon 1, 69100 Villeurbanne, France; 8 Department of Ophthalmology, Universidad Maimónides, Buenos Aires C1405BCK, Argentina; davidpelayes@gmail.com

**Keywords:** diabetic retinopathy, diabetic macular edema, anti-VEGF, precision medicine, optical coherence tomography, OCT angiography, imaging biomarkers, corticosteroids, disease modification

## Abstract

Diabetic retinal disease (DRD) remains one of the leading causes of preventable vision loss worldwide and continues to represent a major global public health challenge despite remarkable therapeutic advances over the past two decades. Intravitreal anti-vascular endothelial growth factor (VEGF) therapy has revolutionized the management of diabetic macular edema and proliferative diabetic retinopathy, significantly improving visual outcomes and reducing vision-threatening complications. Nevertheless, a substantial proportion of patients exhibit incomplete anatomical or functional responses, require frequent lifelong intravitreal injections, or experience disease progression despite adequate VEGF suppression, underscoring the biological heterogeneity of DRD. Growing evidence recognizes DRD as a chronic neurovascular disorder extending beyond a purely microvascular disease. Inflammation, oxidative stress, mitochondrial dysfunction, neurodegeneration, vascular instability, and progressive retinal ischemia interact throughout disease progression. Concurrently, advances in multimodal retinal imaging—including optical coherence tomography (OCT), OCT angiography (OCTA), ultra-widefield imaging, and artificial intelligence-assisted image analysis—have enabled the identification of imaging biomarkers that improve diagnosis, prognostication, and individualized therapeutic decision-making. The therapeutic landscape has expanded considerably beyond conventional anti-VEGF therapy. Corticosteroid implants, dual-pathway inhibitors, sustained drug-delivery systems, vitreoretinal surgery, laser photocoagulation, and emerging pharmacological approaches have broadened treatment options. Increasing evidence supports tailoring therapy according to retinal phenotype, imaging biomarkers, inflammatory status, ischemic burden, and systemic metabolic factors rather than relying on uniform treatment algorithms. This review summarizes recent advances in the understanding and management of DRD, emphasizing pathophysiology, multimodal imaging biomarkers, pharmacological therapies and delivery systems, vitreoretinal surgery, systemic risk-factor optimization, artificial intelligence, and emerging therapeutic strategies. Finally, we introduce Integrated Precision Care (IPC) as a proposed conceptual and research framework that integrates conventional disease staging with multimodal retinal imaging, biological phenotyping, systemic risk assessment, treatment burden, and patient-specific characteristics. IPC is not intended to replace established stage-specific guidelines or to function as a prospectively validated therapeutic algorithm; rather, it provides a hypothesis-generating structure for testing whether multidimensional integration can improve risk stratification, therapeutic selection, durability, patient-centered outcomes, and long-term vision.

## 1. Introduction

Diabetic retinal disease (DRD), encompassing diabetic retinopathy (DR) and diabetic macular edema (DME), remains one of the leading causes of preventable vision loss among working-age adults worldwide and represents one of the most common microvascular complications of diabetes mellitus (DM) [1,2]. The global increase in diabetes prevalence, driven by population aging, obesity, sedentary lifestyles, and longer life expectancy, has resulted in a substantial rise in the number of individuals at risk of developing vision-threatening retinal complications [1,2]. Despite remarkable advances in systemic diabetes management, DRD continues to impose a considerable clinical, economic, and societal burden on healthcare systems worldwide [1,2].

Over the past four decades, the management of DRD has undergone a remarkable transformation. For many years, laser photocoagulation (LP) represented the standard of care for both proliferative diabetic retinopathy (PDR) and DME, primarily aiming to reduce the risk of severe vision loss rather than restore visual function [3]. The introduction of intravitreal anti-vascular endothelial growth factor (VEGF) therapy revolutionized retinal care by providing substantial improvements in visual acuity, superior anatomical outcomes, and effective regression of retinal neovascularization [4]. More recently, corticosteroid implants, dual-pathway inhibition, sustained drug-delivery systems, advances in vitreoretinal surgery, and continuous improvements in retinal imaging have further expanded the therapeutic armamentarium available to retina specialists [5,6]. This evolution is summarized in Table 1.

Although anti-VEGF therapy remains the cornerstone of modern treatment, growing evidence indicates that DRD cannot be fully explained—or adequately managed—through VEGF inhibition alone. A considerable proportion of patients exhibit persistent retinal edema, incomplete visual recovery, treatment resistance, recurrent disease, or substantial treatment burden despite appropriate anti-VEGF therapy [5,7]. These observations underscore the biological heterogeneity of DRD and suggest that multiple pathological mechanisms contribute simultaneously to disease initiation, progression, and therapeutic response [1,5,8].

Current understanding recognizes DRD as a chronic neurovascular disorder involving complex interactions among vascular dysfunction, inflammation, oxidative stress, mitochondrial damage, neurodegeneration, impaired retinal perfusion, and disruption of the blood-retinal barrier. Rather than acting independently, these mechanisms interact dynamically throughout disease progression, amplifying retinal injury and contributing not only to vascular leakage and ischemia but also to irreversible neuronal damage that may precede clinically detectable microvascular abnormalities. Consequently, the traditional vascular-centered model has progressively evolved toward a broader neurovascular paradigm [1,9,10].

Concurrent advances in retinal imaging have transformed diagnosis and monitoring. Optical coherence tomography (OCT), OCT angiography (OCTA), ultra-widefield (UWF) imaging, and artificial intelligence (AI)-assisted image analysis provide complementary structural and vascular information. Importantly, most imaging biomarkers in DRD currently have stronger prognostic than treatment-predictive evidence; their use for selecting a specific therapy generally remains investigational unless a differential treatment effect has been demonstrated.

The therapeutic landscape has also expanded beyond conventional anti-VEGF therapy. Intravitreal corticosteroids, dual-pathway inhibitors targeting VEGF and angiopoietin-2 (Ang-2), sustained drug-delivery systems, refined laser strategies, vitreoretinal surgery, systemic metabolic optimization, and several emerging pharmacological approaches are redefining the management of DRD. Therapeutic decisions are increasingly guided not only by disease severity but also by retinal phenotype, imaging biomarkers, inflammatory status, ischemic burden, vitreoretinal interface abnormalities, lens status, systemic comorbidities, and anticipated treatment adherence [5,6,11].

Collectively, these developments support a more individualized approach while preserving established disease-stage pathways. Contemporary management should first identify the conventional clinical manifestation and indication—DME, NPDR, or PDR—and then use imaging, systemic factors, treatment history, safety, access, adherence, and patient preferences to refine care. IPC is proposed as an overlay on evidence-based management, not as a replacement for it.

The purpose of this review is to provide a comprehensive and contemporary overview of DRD, highlighting recent advances in pathophysiology, multimodal imaging biomarkers (MIB), pharmacological therapies and intraocular drug delivery, vitreoretinal surgery, systemic risk-factor optimization, AI, and emerging therapeutic strategies. We introduce IPC as a proposed conceptual and research framework that integrates these domains while explicitly distinguishing established clinical evidence from emerging phenotype-treatment hypotheses.

The management of DRD has evolved from anatomically directed interventions toward biologically targeted therapies and increasingly durable drug-delivery strategies. This evolution should not be equated automatically with disease modification. In this review, disease control, treatment durability, and disease modification are treated as distinct concepts, and the latter remains an investigational objective requiring durable alteration of the natural disease trajectory.

**Figure 1:**
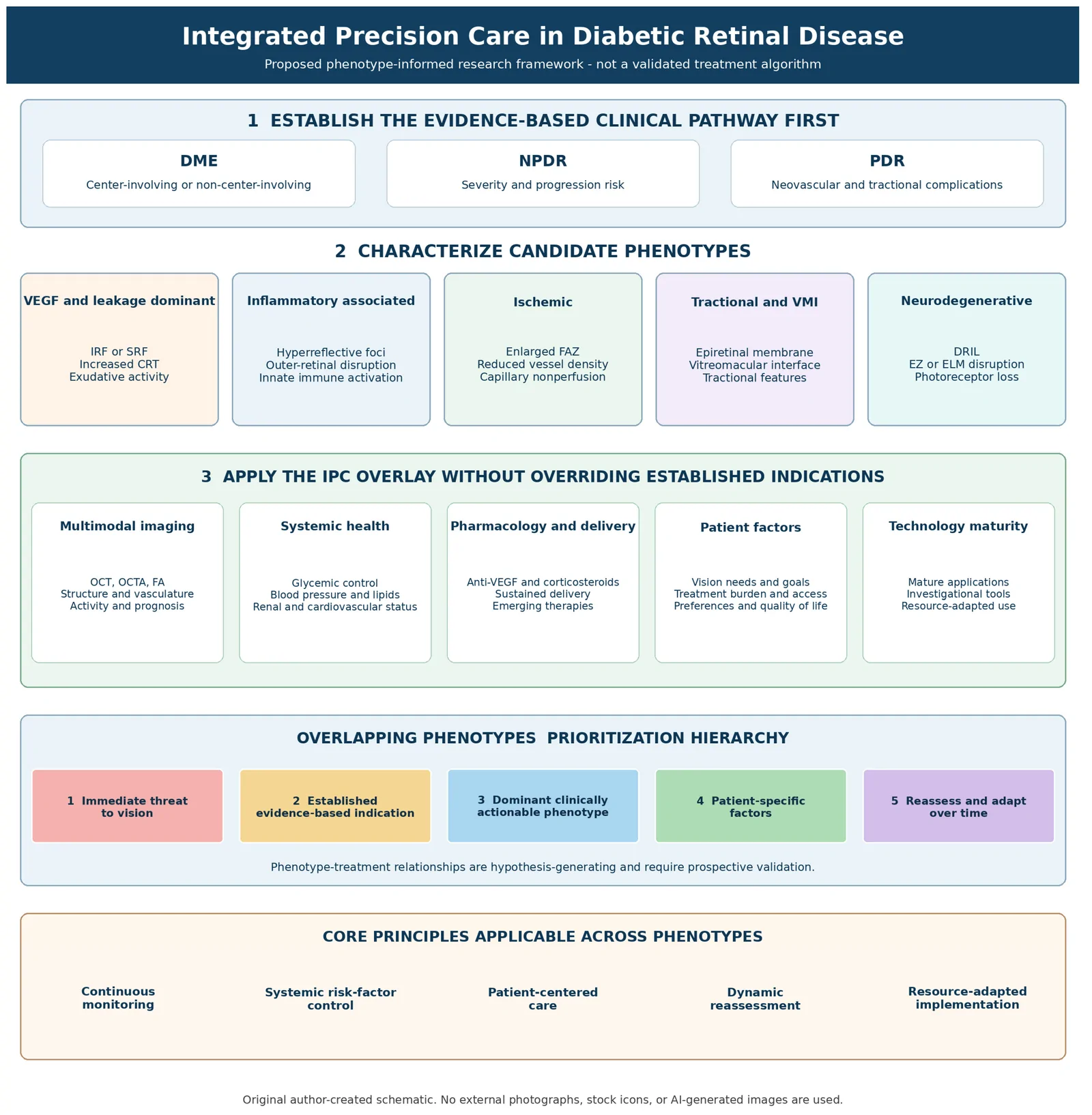
Integrated Precision Care in diabetic retinal disease: proposed phenotype-informed research framework. The revised figure is an original author-created schematic composed exclusively of text and geometric elements; it contains no external photographs, stock icons, or AI-generated images. The framework integrates conventional clinical staging, candidate phenotypes, multimodal imaging, systemic and patient-specific factors, pharmacology and drug delivery, and technology maturity. Phenotype–treatment relationships are research hypotheses unless supported by established clinical indications. The framework should not be interpreted as a prospectively validated clinical treatment algorithm. AI, artificial intelligence; CRT, central retinal thickness; DME, diabetic macular edema; DRIL, disorganization of the retinal inner layers; ELM, external limiting membrane; EZ, ellipsoid zone; FA, fluorescein angiography; FAZ, foveal avascular zone; HRF, hyperreflective foci; IPC, Integrated Precision Care; IRF, intraretinal fluid; NPDR, nonproliferative diabetic retinopathy; OCT, optical coherence tomography; OCTA, optical coherence tomography angiography; PDR, proliferative diabetic retinopathy; PRP, panretinal photocoagulation; SRF, subretinal fluid; VEGF, vascular endothelial growth factor; VMI, vitreomacular interface.

The overall IPC framework proposed throughout this review is summarized in Figure 1. The framework integrates conventional disease staging, candidate phenotypes, multimodal imaging, systemic and patient-specific factors, evidence-supported treatment options, and research priorities. Phenotype–treatment relationships shown in the framework are hypothesis-generating unless supported by established stage-specific indications.

### Stage- and Manifestation-Specific Clinical Pathways

IPC should be applied only after the conventional clinical pathway has been established. In vision-impairing center-involved DME, treatment is guided by visual acuity, center involvement, OCT-defined activity, prior response, ocular status, and evidence-supported pharmacologic options; anti-VEGF therapy remains the usual first-line treatment, with corticosteroids, laser, or surgery used in selected contexts. In NPDR, management is driven by retinopathy severity, presence or absence of DME, progression risk, systemic optimization, and the balance between observation and intervention in selected high-risk eyes. In PDR, neovascular activity, risk of hemorrhage or traction, ability to maintain follow-up, and surgical anatomy determine the use of PRP, anti-VEGF therapy, and vitrectomy. IPC therefore complements—rather than supersedes—these established DME, NPDR, and PDR pathways.

## 2. Modern Understanding of Diabetic Retinal Disease: Beyond a Vascular Disorder

For decades, DR was regarded primarily as a microvascular complication of DM, characterized by progressive capillary damage, increased vascular permeability, retinal ischemia, and pathological neovascularization. This vascular-centered paradigm successfully explained many of the clinical manifestations observed during advanced stages of the disease and provided the biological foundation for retinal LP and, subsequently, intravitreal anti-VEGF therapy. However, despite the remarkable success of these treatments, accumulating clinical and experimental evidence indicates that vascular dysfunction alone cannot fully explain the marked heterogeneity in disease progression, visual outcomes, or therapeutic response [1,10,12].

Current evidence supports a broader concept in which DRD is recognized as a disorder of the retinal neurovascular unit. This highly integrated functional unit comprises retinal endothelial cells, pericytes, Müller cells, astrocytes, microglia, neurons, and the retinal pigment epithelium, all of which interact continuously to preserve retinal homeostasis. Chronic hyperglycemia disrupts these tightly coordinated cellular interactions through oxidative stress, mitochondrial dysfunction, activation of inflammatory pathways, accumulation of advanced glycation end-products, and metabolic dysregulation. Rather than occurring sequentially, these pathogenic mechanisms develop simultaneously, interact dynamically, and reinforce one another, ultimately driving progressive structural and functional retinal damage [1,10,13,14,15].

One of the most significant advances in recent years has been the recognition that neurodegeneration is an early event in DRD. Functional abnormalities—including reduced contrast sensitivity, impaired dark adaptation, color vision deficits, and electrophysiological alterations—have been documented before clinically detectable vascular lesions become apparent. Likewise, OCT has demonstrated thinning of the ganglion cell layer and inner retinal layers in diabetic individuals without clinically evident retinopathy, suggesting that neuronal injury may precede, rather than simply result from, microvascular damage. This paradigm has fundamentally reshaped our understanding of disease pathogenesis and has stimulated increasing interest in neuroprotective therapeutic strategies [1,14,16,17].

Chronic inflammation has also emerged as a central driver of disease progression. Persistent hyperglycemia promotes activation of retinal microglia and Müller cells, leading to the release of numerous pro-inflammatory cytokines, chemokines, and adhesion molecules that contribute to leukostasis, endothelial dysfunction, disruption of the blood-retinal barrier, and increased vascular permeability. Importantly, inflammatory mediators interact synergistically with VEGF, amplifying tissue injury and helping explain why a substantial proportion of patients exhibit incomplete or transient responses to VEGF inhibition. These observations provide a strong biological rationale for incorporating anti-inflammatory therapies into the management of selected patients [10,13,18,19].

Although VEGF remains a pivotal mediator of DME and retinal neovascularization, it represents only one component of a complex molecular network. Additional pathways—including angiopoietin/Tie2 signaling, complement activation, oxidative stress, extracellular matrix remodeling, mitochondrial dysfunction, and progressive retinal ischemia—contribute substantially to disease progression. The relative contribution of these mechanisms varies among individuals and changes throughout the course of the disease, reinforcing the concept that DRD is biologically heterogeneous and unlikely to respond optimally to a uniform therapeutic strategy [1,2,10,20,21].

The rapid evolution of multimodal retinal imaging has transformed diagnosis, staging, monitoring, and prognostication in DRD. However, an important distinction is required between prognostic and predictive biomarkers. Prognostic biomarkers provide information about expected disease course or clinical outcome irrespective of a specific intervention, whereas predictive biomarkers identify a differential likelihood of benefit or harm from a particular treatment and therefore require evidence of a biomarker–treatment interaction. Most current OCT and OCTA biomarkers in DRD are better supported as prognostic or phenotypic markers than as validated treatment-selection tools.

## 3. Imaging Biomarkers: The Bridge Between Biology and Precision Therapy

DRIL is among the most reproducible structural markers associated with visual function. Greater DRIL is associated with worse visual acuity and poorer functional prognosis, but it should currently be regarded primarily as a prognostic biomarker rather than a validated marker for selecting a specific ocular therapy [22,23,24,25,26,27,28].

Similarly, disruption of the external limiting membrane or ellipsoid zone is associated with reduced visual potential and poorer functional outcomes. These outer-retinal features are clinically useful for prognosis and counseling, but they do not currently provide a validated basis for choosing one pharmacologic agent over another [29].

Hyperreflective retinal foci (HRF), large intraretinal cysts, subretinal fluid, vitreomacular-interface abnormalities, and choroidal changes have all been investigated as candidate biomarkers. HRF may be associated with inflammatory activity, but available evidence does not establish a validated HRF threshold or demonstrate that HRF alone prospectively identifies patients who derive greater benefit from corticosteroids than from anti-VEGF therapy. Association with outcome after treatment should not be interpreted as predictive utility without evidence of a differential treatment effect [30,31,32,33,34].

OCTA provides noninvasive visualization of retinal microvasculature and can quantify the foveal avascular zone, vessel density, and capillary nonperfusion. These measures may contribute to prognosis and risk stratification, but device, acquisition, segmentation, and population heterogeneity currently preclude universal treatment-selection cutoffs. Retinal nonperfusion should therefore be distinguished from proliferative disease and does not, by itself, constitute an indication for PRP or anti-VEGF therapy [35].

The principal MIBs currently used in DRD are summarized in Table 2. The table now distinguishes biological or clinical association, current biomarker role, and therapeutic implication. This distinction is intended to prevent associative imaging findings from being interpreted as validated treatment-selection rules.

Collectively, multimodal imaging supports phenotypic characterization, prognosis, longitudinal monitoring, and recognition of clinically actionable anatomy. Its incremental value for selecting a specific pharmacologic therapy remains incompletely validated for many biomarkers. Prospective biomarker-stratified studies are required before most imaging features can be used as treatment-predictive markers within IPC.

## 4. Anti-VEGF Therapy: Current Role, Limitations, and Future Perspectives

The magnitude of anti-VEGF benefit is well established in pivotal randomized trials. In RISE and RIDE, 33.6–44.8% of eyes receiving ranibizumab 0.3 mg gained at least 15 ETDRS letters at 24 months, compared with 12.3–18.1% of sham-treated eyes. In DRCR.net Protocol T, mean 1-year visual-acuity gains were +13.3, +9.7, and +11.2 letters with aflibercept, bevacizumab, and ranibizumab, respectively; among eyes presenting with 20/50 or worse vision, gains were +18.9, +11.8, and +14.2 letters. More recent trials have extended durability: YOSEMITE/RHINE demonstrated noninferior visual outcomes with faricimab using intervals up to 16 weeks, while PHOTON showed that aflibercept 8 mg maintained visual and anatomic benefit through 96 weeks with fewer injections than aflibercept 2 mg every 8 weeks. These data establish anti-VEGF therapy as the evidence-based foundation for vision-impairing center-involved DME while demonstrating clinically relevant heterogeneity in response and durability. Selected quantitative evidence from pivotal and contemporary DME trials is summarized in Table 3 [36].

VEGF is a central mediator of vascular permeability, angiogenesis, and endothelial dysfunction in DRD. Chronic hyperglycemia, retinal hypoxia, oxidative stress, and inflammation stimulate VEGF expression by multiple retinal cell populations, resulting in disruption of the blood-retinal barrier, increased vascular leakage, endothelial proliferation, and pathological neovascularization. Pharmacological inhibition of VEGF effectively interrupts these processes, leading to rapid reduction in macular edema, stabilization of the retinal microvasculature, and regression of neovascular tissue. Established anti-VEGF agents—including bevacizumab, ranibizumab, aflibercept, and faricimab—have demonstrated substantial efficacy, while newer long-acting strategies continue to expand durability options. VEGF inhibition therefore remains the therapeutic foundation for many patients with vision-threatening DRD [1,7,37,38,39,40].

Treatment burden remains a major challenge. Frequent intravitreal injections and monitoring visits impose direct and indirect costs on patients, caregivers, clinicians, and healthcare systems. Pharmacologic durability should therefore be considered alongside efficacy, safety, access, adherence, and patient preference. Longer dosing intervals may reduce burden, but durability alone does not establish superior patient-centered or economic value [41,42,43,44].

Recent innovations include dual VEGF-A/Ang-2 inhibition with faricimab, higher-dose aflibercept, brolucizumab, and continuous or sustained intraocular delivery. KESTREL/KITE demonstrated durable anatomic and visual outcomes with brolucizumab but also confirmed a drug-specific inflammatory safety signal, including intraocular inflammation, retinal vasculitis, and retinal vascular occlusion. Real-world DME studies of faricimab, including FARETINA-DME and J-CREST, support effectiveness and interval extension in routine practice but should not be interpreted as randomized comparative efficacy. The Port Delivery System with ranibizumab has also demonstrated noninferior visual outcomes to monthly ranibizumab in DME with refill-exchange every 24 weeks, illustrating how formulation and delivery technology can alter exposure and treatment burden [45,46,47,48,49].

Safety considerations must be balanced across modalities. Anti-VEGF therapy generally has a favorable ocular safety profile, although repeated injections carry small cumulative risks of endophthalmitis, intraocular inflammation, retinal tear or detachment, and transient intraocular-pressure elevation. Corticosteroids provide broader anti-inflammatory activity and longer exposure but increase the risks of cataract and ocular hypertension. Laser may produce permanent retinal scars and, depending on treatment pattern, field or night-vision effects. Sustained-release or refillable systems introduce device- and procedure-specific risks, while vitrectomy carries established intraoperative and postoperative complications. Individualized treatment selection should therefore integrate efficacy, durability, ocular status, systemic vascular risk, treatment burden, and modality-specific safety rather than efficacy alone.

Recent therapeutic innovations illustrate the continuing evolution of VEGF-targeted therapy. Faricimab, through simultaneous inhibition of VEGF-A and Ang-2, represents the first bispecific antibody approved for DME and offers the potential for longer treatment intervals in appropriately selected patients. Additional strategies—including high-dose aflibercept, sustained drug-delivery platforms, gene therapy, and next-generation intraocular delivery systems—seek to preserve visual outcomes while reducing treatment burden. Although these approaches are promising, their long-term effectiveness, durability, cost-effectiveness, and real-world performance remain areas of active investigation [2,7,21,50,51,52,53].

Increasing evidence supports a more individualized approach to VEGF inhibition. Rather than considering anti-VEGF therapy as a universal solution for all patients, treatment decisions should integrate MIB, retinal phenotype, inflammatory activity, ischemic burden, systemic metabolic status, previous therapeutic response, and anticipated treatment adherence. Such an approach recognizes that anti-VEGF therapy remains the cornerstone of DRD management while acknowledging that many patients may benefit from complementary or alternative therapeutic strategies. Ultimately, the role of VEGF inhibition is evolving from a one-size-fits-all treatment toward a component of a broader, personalized therapeutic strategy. This evolution provides the clinical foundation for IPC, in which treatment selection is driven by the underlying biology of disease rather than by VEGF activity alone [1,2,11].

## 5. Pharmaceutical Formulations, Intraocular Drug Delivery, and Pharmacokinetic/Pharmacodynamic Considerations

For posterior-segment disease, therapeutic efficacy depends not only on molecular target but also on formulation, route of administration, intraocular distribution, clearance, and the duration for which retinal drug exposure remains above a biologically effective threshold. Intravitreal administration bypasses the corneal, conjunctival, blood-aqueous, and blood-retinal barriers that limit posterior exposure after topical or systemic dosing. Nevertheless, intravitreal molecules are cleared through anterior and/or posterior pathways, and ocular half-life, molecular size, binding affinity, dose, vitreous status, and target-mediated disposition can influence duration of pharmacologic activity.

For anti-VEGF biologics, pharmacokinetic half-life should not be equated directly with clinical dosing interval. Pharmacodynamic durability also depends on molar dose, binding potency, target turnover, tissue distribution, and the concentration required for sustained pathway suppression. Faricimab population pharmacokinetic modeling estimated a vitreous half-life of approximately 7.5 days and demonstrated that dosing frequency could not be explained by vitreous half-life alone. Similarly, increasing the delivered dose, as with aflibercept 8 mg, can prolong the period of effective VEGF suppression without requiring a fundamentally different target. These principles help explain why agents with broadly similar mechanisms can support different treatment intervals [54,55].

Corticosteroid formulations illustrate more directly how drug-delivery design changes intraocular exposure. Soluble or suspended triamcinolone provides finite intravitreal residence, whereas the biodegradable dexamethasone implant produces a high early exposure followed by declining release over several months. The non-bioerodible fluocinolone acetonide implant provides low-dose continuous release, approximately 0.2 micrograms/day, for up to three years. These distinct exposure profiles affect retreatment frequency, cumulative steroid exposure, and the temporal pattern of adverse effects such as ocular hypertension and cataract [56,57].

Sustained and continuous anti-VEGF delivery seeks to reduce peak-trough fluctuation and treatment burden. Refillable reservoir systems can maintain intraocular drug exposure over months but exchange repeated needle procedures for an implanted device and refill-exchange procedures, creating a different safety and logistical profile. The PAGODA trial demonstrated that continuous ranibizumab delivery with refill-exchange every 24 weeks produced visual outcomes comparable to monthly ranibizumab through 64 weeks in DME, while device/procedure-related adverse events require specific consideration [48,49].

Alternative posterior-segment routes are also being investigated. Suprachoroidal delivery can preferentially expose choroidal and retinal tissues while potentially reducing anterior-segment and systemic exposure, although its role in routine DME care remains incompletely established. Polymeric depots, hydrogels, nanoparticles, microparticles, and gene-based approaches aim to extend exposure or provide continuous intraocular production of therapeutic proteins. These technologies remain heterogeneous in maturity and should be evaluated according to release kinetics, biocompatibility, dose reproducibility, reversibility, manufacturability, device-related complications, and the ability to demonstrate clinically meaningful benefit beyond reduced injection frequency [58].

Within IPC, PK/PD and formulation characteristics should therefore be treated as patient- and phenotype-relevant variables rather than technical details. A longer-acting formulation may be particularly valuable when adherence, travel, caregiver dependence, or recurrent disease makes frequent treatment impractical, whereas reversibility and shorter exposure may be preferable when safety uncertainty is high. Precision care should match not only the biological target but also the exposure profile and delivery platform to the patient’s ocular status, prior response, safety risk, and real-world capacity to sustain treatment.

## 6. Corticosteroids: The Role of Inflammation in Precision Therapy

Although VEGF plays a central role in the pathogenesis of DME, growing evidence indicates that chronic inflammation is an equally important driver of disease progression. Persistent hyperglycemia induces activation of retinal microglia, Müller cells, endothelial cells, and other components of the retinal neurovascular unit, resulting in sustained production of pro-inflammatory cytokines, chemokines, prostaglandins, adhesion molecules, and additional inflammatory mediators. These processes contribute to disruption of the blood-retinal barrier, increased vascular permeability, leukostasis, neuronal dysfunction, and progressive retinal injury. Unlike VEGF, which predominantly mediates vascular leakage and angiogenesis, inflammatory pathways affect multiple cellular components simultaneously and may persist despite adequate VEGF suppression. This biological complexity provides a strong rationale for incorporating intravitreal corticosteroids into the management of appropriately selected patients with DRD [1,12,13,18,59].

Corticosteroids exert broad anti-inflammatory effects by modulating multiple pathogenic pathways simultaneously. They suppress the expression of pro-inflammatory cytokines, reduce leukocyte adhesion, stabilize the blood-retinal barrier, decrease vascular permeability, inhibit VEGF production, and promote resolution of retinal edema. In addition, corticosteroids may attenuate Müller cell activation, regulate microglial responses, and reduce secondary neuronal injury, suggesting therapeutic effects that extend beyond fluid resorption alone. By targeting several interconnected biological mechanisms, corticosteroids should be regarded as a complementary therapeutic strategy rather than simply an alternative to VEGF inhibition [1,12,13,18,59].

The development of sustained-release intravitreal implants has improved corticosteroid durability. The biodegradable dexamethasone implant has demonstrated efficacy in DME and is used in selected eyes in which anti-VEGF response, injection burden, lens status, prior steroid response, vitrectomy status, or other clinical considerations favor corticosteroid therapy. Its well-characterized safety profile requires monitoring for ocular hypertension and cataract progression.

The fluocinolone acetonide intravitreal implant represents a complementary long-term therapeutic strategy by providing continuous low-dose corticosteroid release for up to several years. This sustained-delivery approach is particularly attractive for patients with chronic, recurrent DME requiring repeated intravitreal therapy. By substantially reducing treatment frequency, fluocinolone may decrease treatment burden while maintaining long-term anatomical stability. Nevertheless, careful patient selection remains essential because prolonged corticosteroid exposure is associated with an increased risk of cataract progression and ocular hypertension, necessitating regular clinical monitoring [60,61,62].

Appropriate patient selection is central to corticosteroid use. Persistent or recurrent DME, pseudophakia, previous therapeutic response, difficulty adhering to frequent injections, and contraindications or limitations of anti-VEGF therapy may support consideration of a steroid implant. Imaging features such as HRF may be associated with inflammatory activity, but no single OCT biomarker should currently be used in isolation to select corticosteroid therapy, and biomarker-guided steroid superiority remains unvalidated [63,64,65].

The safety profile of intravitreal corticosteroids is well established. Cataract progression and intraocular pressure elevation remain the two principal adverse events. Fortunately, both complications are generally predictable and manageable through appropriate follow-up, topical intraocular pressure-lowering medications, selective laser or surgical intervention when necessary, and cataract extraction when indicated. Importantly, numerous studies have demonstrated that the visual benefits achieved after cataract surgery frequently outweigh the temporary reduction in lens transparency associated with corticosteroid treatment. Therefore, the possibility of cataract development should not, by itself, preclude corticosteroid therapy in appropriately selected patients [56,60,61,62].

Corticosteroids are an established therapeutic option for selected patients with DME, but their placement should remain individualized rather than dictated by a presumed inflammatory imaging phenotype. Lens status, glaucoma or steroid-response history, prior anti-VEGF response, treatment burden, and patient preference should be integrated. Whether phenotype-guided corticosteroid selection improves outcomes beyond conventional clinical selection requires prospective evaluation. The randomized Protocol B comparison and its three-year follow-up provide key long-term evidence for intravitreal triamcinolone versus focal/grid laser [66,67].

Selected quantitative evidence from pivotal corticosteroid trials is summarized in Table 4.

**Table 4:** Selected quantitative evidence from pivotal corticosteroid trials in DME, including sample size, regimen and follow-up, visual and anatomic outcomes, treatment burden or durability, and key safety information.

Trial	N	Regimen/Follow-Up	Visual Outcome	Anatomic Outcome	Treatment Burden/Durability	Key Safety Note
DRCR.net Protocol B (Ip et al., 2008) [66]	840 eyes/693 subjects	Intravitreal triamcinolone 1 mg or 4 mg vs. focal/grid laser; retreatment q4mo PRN; 2–3 yr follow-up	No VA benefit over laser at 2 yr; by 3 yr, laser eyes gained +5 letters vs. 0 letters in both triamcinolone arms	Early (4 mo) reduction in retinal thickening with triamcinolone paralleled the VA findings but was not sustained	Repeat injections q4mo; laser remained the more durable, lower-risk benchmark	Cataract surgery in 13%/23%/51% (laser/1 mg/4 mg) by 2 yr, rising to 83% in the 4 mg arm by 3 yr; IOP rise ≥ 10 mmHg in 4%/16%/33%
DRCR.net Protocol I (Elman et al., 2010, 2015) [68,69]	854 eyes/691 participants	Triamcinolone 4 mg + prompt laser vs. ranibizumab + prompt/deferred laser vs. sham + prompt laser; 2 yr	Triamcinolone + laser underperformed ranibizumab overall, but in pseudophakic eyes, VA gains approached the ranibizumab arms with fewer injections	Comparable reduction in central subfield thickness across active treatment arms	Fewer intravitreal injections than ranibizumab regimens, but paired with prompt laser	Substantially higher rates of cataract progression and IOP elevation with triamcinolone than with ranibizumab
MEAD (Boyer et al., 2014) [56]	1048 patients	Dexamethasone implant (Ozurdex) 0.7 mg or 0.35 mg vs. sham; retreatment no more than q6mo; 3 yr	≥15-letter BCVA gain at study end in 22.2% (0.7 mg) vs. 12.0% (sham)	Significant, sustained reduction in central retinal thickness vs. sham	Median of ~4–5 injections over 3 years (dosing interval ~6 mo)	Cataract in ~68% of phakic 0.7 mg eyes; IOP rise ≥ 10 mmHg in ~27–32%, generally controlled with topical drops
FAME A/B (Campochiaro et al., 2012) [60]	956 patients (pooled)	Fluocinolone acetonide implant (Iluvien) 0.2 ug/day or 0.5 ug/day vs. sham; single injection; 24–36 mo	≥15-letter BCVA gain in the chronic-DME subgroup: ~34% (low dose) vs. ~13% (sham) at 36 mo	Sustained reduction in central retinal/foveal thickness maintained through 36 months	Single implant releasing drug for up to 36 months; low re-injection rate versus repeat-dosed agents	Cataract surgery in >80% of phakic eyes; incisional glaucoma surgery required in ~5–8% for IOP elevation

Abbreviations. BCVA = best-corrected visual acuity; DME = diabetic macular edema; IOP = intraocular pressure; PRN = pro re nata; VA = visual acuity.

## 7. Laser Therapy: An Evolving Role Rather than an Obsolete Treatment

The introduction of intravitreal anti-VEGF therapy profoundly transformed the role of LP in DRD. For several decades, focal/grid laser for DME and panretinal photocoagulation (PRP) for PDR represented the standard of care, supported by landmark clinical trials demonstrating substantial reductions in the risk of severe vision loss. With the widespread adoption of anti-VEGF therapy, however, laser treatment evolved from a primary therapeutic modality to a more selective and complementary intervention. This transition has led to the misconception that LP has become obsolete. Instead, its role has become more individualized and biologically targeted [1,3,68,69,70].

Unlike pharmacological therapies, whose effects depend on sustained intraocular drug concentrations, LP produces durable structural and physiological changes within the retina. Focal or grid laser promotes selective closure of leaking microaneurysms, reduces retinal oxygen demand, stabilizes the blood-retinal barrier, and decreases chronic fluid accumulation. Similarly, PRP reduces ischemic drive by decreasing metabolic demand in the peripheral retina, thereby lowering intraocular VEGF production and promoting regression of retinal neovascularization. These mechanisms differ fundamentally from pharmacological VEGF inhibition and illustrate how laser and intravitreal therapies target complementary aspects of disease pathophysiology [3,70,71].

Randomized clinical trials have consistently demonstrated superior visual outcomes with anti-VEGF therapy compared with laser monotherapy for center-involving DME. However, these findings should not be interpreted as evidence against laser therapy itself. Rather, they emphasize that different therapeutic modalities address distinct biological mechanisms. Whereas anti-VEGF agents primarily suppress vascular permeability and angiogenesis, laser therapy modifies retinal oxygen consumption, ischemic burden, and long-term vascular remodeling. Consequently, these strategies should be considered complementary rather than mutually exclusive [4,7,37,38,39,40,69,72].

In contemporary clinical practice, LP continues to play an important role in carefully selected clinical scenarios. Focal laser remains an effective option for non-center-involving DME associated with well-defined leaking microaneurysms, particularly when visual acuity is preserved. Likewise, PRP remains a highly effective treatment for PDR, especially in patients with limited access to regular follow-up, anticipated poor adherence to repeated intravitreal injections, or contraindications to long-term anti-VEGF therapy. Combination therapy using anti-VEGF agents together with PRP has demonstrated favorable outcomes by achieving rapid regression of retinal neovascularization while providing durable control of ischemic retinal disease [39,69,73,74].

Technological innovations have further refined the role of laser therapy. Pattern-scanning laser systems, navigated laser platforms, subthreshold micropulse laser, and endpoint management technologies seek to maximize therapeutic efficacy while minimizing collateral retinal damage. Among these advances, subthreshold micropulse laser has generated particular interest because it delivers repetitive low-energy pulses that stimulate retinal pigment epithelium function without producing visible retinal burns. Although its precise biological mechanisms remain incompletely understood, this approach may reduce retinal edema while preserving retinal architecture, making it an attractive option for selected patients [75,76,77,78,79,80].

Contemporary management increasingly favors individualized laser application rather than fixed treatment algorithms. The decision to perform laser therapy should incorporate edema location, retinal ischemia, neovascular activity, MIB, anticipated treatment adherence, systemic health, socioeconomic circumstances, and previous therapeutic response. In selected patients, laser therapy may also reduce treatment burden by decreasing the frequency of intravitreal injections or consolidating disease stability after pharmacological control has been achieved [2,11,73,78,81].

Within the framework of IPC, LP should no longer be viewed as either first-line therapy or an outdated intervention. Instead, it should be recognized as an integral component of a multimodal therapeutic strategy in which each treatment modality addresses different biological mechanisms and clinical needs. Appropriate patient selection, guided by retinal phenotype, imaging biomarkers, and individual patient characteristics, remains the principal determinant of successful outcomes. As our understanding of DRD continues to evolve, laser therapy is expected to maintain a selective but essential role within personalized management algorithms [2,73,75].

## 8. Vitrectomy in Diabetic Retinal Disease: Beyond Mechanical Intervention

Pars plana vitrectomy (PPV) remains an established treatment for advanced complications of diabetic retinopathy, including non-clearing vitreous hemorrhage, tractional retinal detachment involving or threatening the macula, combined tractional-rhegmatogenous retinal detachment, and selected cases of severe fibrovascular proliferation or clinically significant vitreomacular traction. These established indications should be distinguished from the emerging concept of earlier phenotype-guided surgery in eyes without conventional surgical indications.

The biological rationale for vitrectomy extends far beyond the removal of vitreous opacities or tractional membranes. The vitreous cavity functions as a reservoir for VEGF, inflammatory cytokines, chemokines, growth factors, and other mediators involved in the pathogenesis of DRD. Vitrectomy facilitates the elimination of these molecules, enhances intraocular oxygen diffusion from the anterior segment to the posterior pole, improves retinal oxygenation, and may reduce the chronic ischemic stimulus responsible for sustained VEGF production. Collectively, these physiological changes suggest that vitrectomy favorably modifies the retinal microenvironment through mechanisms that extend well beyond mechanical traction release [1,82,83].

The role of vitrectomy in DME remains an area of active investigation. Eyes with clinically significant vitreomacular traction consistently benefit from surgical release, frequently resulting in anatomical improvement and, in appropriately selected cases, meaningful visual recovery. Increasing evidence also suggests that more subtle abnormalities of the vitreomacular interface—including epiretinal membranes, incomplete posterior vitreous detachment, persistent posterior hyaloid attachment, or reduced retinal compliance—may contribute to chronic edema in a subset of patients. Careful interpretation of high-resolution OCT has therefore become essential for identifying surgical candidates, particularly in eyes with persistent DME despite optimized pharmacological therapy [11,24,84,85,86].

Internal limiting membrane (ILM) peeling has become an integral component of vitrectomy in many patients with DRD. Removal of the ILM may eliminate residual tangential traction, reduce the scaffold for subsequent epiretinal membrane formation, improve retinal fluid dynamics, and facilitate more complete retinal remodeling. Although randomized studies have demonstrated variable functional outcomes, ILM peeling appears to reduce recurrence of macular edema and improve long-term anatomical stability in appropriately selected eyes. Nevertheless, the decision to perform ILM peeling should remain individualized according to retinal morphology, surgical objectives, and surgeon experience [85,86].

Timing remains important once a clinically meaningful surgical indication is present. In the systematic review and meta-analysis by McCullough et al., 38 studies including 3839 eyes undergoing PPV for diabetic tractional retinal detachment showed an overall failure rate of retinal reattachment after one surgery of 5.9%, corresponding to approximately 94.1% primary anatomic success. Functional outcomes remained guarded, and better preoperative visual acuity was the principal factor associated with better postoperative vision. These data support timely intervention for established indications before irreversible macular damage becomes advanced; they do not establish prophylactic vitrectomy for biological disease modification [87].

Modern multimodal retinal imaging has become indispensable for surgical decision-making. OCT enables detailed assessment of vitreomacular interface abnormalities, retinal architecture, photoreceptor integrity, retinal thickness, and structural biomarkers associated with postoperative prognosis. OCTA and ultra-WFI further complement this evaluation by characterizing retinal perfusion, ischemic burden, peripheral non-perfusion, and disease extent. Integration of these MIBs with clinical findings allows more accurate patient selection, individualized surgical planning, and realistic prognostic counseling [2,11,88,89,90,91,92,93,94,95].

Within IPC, vitrectomy should retain its established role for recognized surgical indications. Extending surgery to earlier disease stages solely on the basis of inflammatory burden, vitreous cytokine accumulation, retinal oxygenation, or imaging-defined biological phenotypes remains investigational. Although these mechanisms provide biological plausibility, current evidence is insufficient to recommend prophylactic or early vitrectomy for disease modification in the absence of an established surgical indication. Prospective studies are required to determine whether specific phenotypes can improve surgical timing beyond conventional clinical criteria.

## 9. Systemic Disease Modification: Treating the Patient Beyond the Retina

DRD is the ocular manifestation of a complex systemic metabolic disorder rather than an isolated retinal condition. Strong evidence demonstrates that optimization of systemic risk factors reduces the incidence and progression of diabetic retinopathy; however, evidence that systemic optimization directly modifies the efficacy or durability of a specific ocular therapy is less established. Systemic targets within IPC should therefore be interpreted according to contemporary diabetes, cardiovascular, and renal guidelines rather than as retina-specific treatment thresholds.

Among systemic factors, glycemic control remains central. For many nonpregnant adults, an HbA1c target below 7% is appropriate, although goals should be individualized according to age, diabetes duration, comorbidity, hypoglycemia risk, functional status, and life expectancy. Glycemic variability and rapid changes in glycemia may also be clinically relevant, but they should not be converted into unvalidated retina-specific thresholds [96,97,98,99].

Automated insulin delivery (AID) systems can produce rapid and substantial improvements in glycemic control, raising the clinically relevant question of early worsening of diabetic retinopathy (EWDR) after abrupt glucose lowering. Recent prospective observational data in people with type 1 diabetes commencing AID are reassuring: short-term retinal status was generally stable, and subsequent data in adolescents and young adults did not identify an increased EWDR signal attributable to AID despite rapid and large glycemic improvements. These findings support the metabolic benefits of AID while reinforcing the importance of appropriate ophthalmic surveillance when glycemia improves rapidly, particularly in patients with pre-existing retinopathy [100,101].

The principal systemic factors influencing disease progression, retinal phenotype, therapeutic response, and long-term visual outcomes are summarized in Table 5. These variables should be considered integral components of individualized therapeutic planning within the IPC framework [102,103].

Systemic hypertension is another major modifiable determinant of progression. In patients with diabetes and hypertension, a blood-pressure target below 130/80 mmHg is recommended when it can be safely achieved, with individualization according to cardiovascular, renal, and treatment-related factors. Lipid management should follow contemporary cardiovascular-risk-based recommendations rather than a single retinal-specific lipid threshold. Fenofibrate has demonstrated retinopathy-related benefit in selected trial populations but should be considered within overall metabolic and cardiovascular management rather than as a universal retina-directed treatment.

Chronic kidney disease has emerged as one of the strongest systemic predictors of advanced DRD. The retina and kidney share multiple pathophysiological mechanisms, including endothelial dysfunction, chronic inflammation, oxidative stress, and increased vascular permeability. Reduced renal function, albuminuria, and progressive nephropathy have consistently been associated with a higher prevalence of DME, PDR, and poorer visual outcomes. These observations underscore the importance of close collaboration among ophthalmologists, endocrinologists, nephrologists, cardiologists, and primary care physicians to optimize systemic management and improve both ocular and overall health outcomes. The concept of the diabetic renal-retinal syndrome further supports the use of renal biomarkers, including estimated glomerular filtration rate and albuminuria, as systemic indicators that may help stratify retinal disease severity and guide individualized management within the IPC model [104,105].

The emergence of newer glucose-lowering therapies has generated considerable interest regarding their potential influence on DRD. Sodium-glucose cotransporter-2 inhibitors have demonstrated substantial cardiovascular and renal protection, while experimental and observational studies suggest additional anti-inflammatory, endothelial, and microvascular benefits that may also extend to the retina. Likewise, glucagon-like peptide-1 receptor agonists have become central components of modern diabetes management because of their favorable effects on glycemic control, weight reduction, and cardiovascular risk. Although early concerns were raised regarding transient worsening of DR during rapid glycemic improvement, current evidence suggests that long-term retinal outcomes are determined predominantly by the magnitude and stability of metabolic control rather than by direct retinal toxicity of these medications. Ongoing prospective studies will further clarify their role in retinal disease modification [6,106,107].

Lifestyle modification remains one of the most effective—and frequently underestimated—therapeutic interventions. Regular physical activity, weight reduction, smoking cessation, healthy nutrition, adequate sleep, and optimization of cardiovascular risk factors contribute to improved metabolic control, reduced systemic inflammation, and enhanced vascular health. Beyond reducing the incidence and progression of DRD, these interventions improve patients’ ability to adhere to long-term ophthalmic treatment and multidisciplinary care. Accordingly, patient education should be regarded as an integral therapeutic intervention rather than merely supportive counseling [6,108].

Within IPC, the ophthalmologist identifies ocular manifestations and communicates systemic risk, but does not independently manage all systemic targets. Primary-care physicians and endocrinologists or diabetes-care specialists coordinate glycemic and general cardiometabolic management; nephrologists manage clinically significant or progressive kidney disease and albuminuria; and cardiologists contribute when established cardiovascular disease, heart failure, or complex cardiovascular risk requires specialist care. IPC therefore promotes communication and coordinated care rather than transfer of systemic medical management to the retina clinic.

Obesity has emerged as an independent contributor to systemic inflammation, insulin resistance, and endothelial dysfunction. Weight reduction—whether achieved through lifestyle modification, pharmacological therapy, or metabolic surgery—may improve systemic metabolic health and indirectly influence the progression of DRD. Although the direct effects of weight loss on retinal outcomes continue to be investigated, its established cardiovascular and metabolic benefits support its inclusion within comprehensive disease management [108].

### 9.1. Practical Implementation, Accessibility, and Resource Considerations

IPC should be scalable and should not depend on universal access to every advanced technology described in this review. A core level can be implemented using conventional staging, visual acuity, ophthalmic examination, structural OCT when available, systemic risk assessment, treatment history, adherence, and patient preferences. An enhanced level may incorporate OCTA, UWF imaging or angiography, quantitative longitudinal imaging, and structured multidisciplinary coordination.

Advanced or research-level IPC may additionally investigate AI-based multimodal integration, molecular biomarkers, multiomics, foundation models, and digital twins. These research technologies are not prerequisites for individualized care.

Implementation must account for acquisition and maintenance costs, software interoperability, data storage, reimbursement, availability of retina specialists, access to multidisciplinary care, and training. OCTA and quantitative imaging require standardized acquisition and interpretation because segmentation error, device-specific measurements, and image quality can alter biomarker estimates. AI-supported tools additionally require clinician oversight, external validation, data governance, interoperability, and procedures for managing uncertain or discordant outputs.

Accordingly, IPC should be considered resource-adaptable rather than technology-dependent. Increasing technological sophistication should be incorporated only when it is validated, clinically actionable, and economically sustainable, and should not widen disparities in retinal care. Prospective evaluation should therefore include feasibility, accessibility, equity, and health-economic outcomes across different healthcare systems.

### 9.2. Patient-Reported Outcomes, Quality of Life, and Treatment Burden

Clinical and anatomical outcomes alone do not fully capture the impact of DRD or its treatment. Vision-related functioning, treatment satisfaction, injection-related anxiety or discomfort, travel time, clinic attendance, employment disruption, caregiver dependence, financial burden, and ability to maintain daily activities may substantially influence the real-world value of therapy. Patient-reported outcome measures such as the NEI VFQ-25 can complement visual-acuity and anatomical endpoints.

Treatment burden should be considered multidimensional rather than defined solely by injection frequency. In a multinational survey, 66.1% of patients with DME reported at least one treatment-related barrier, and work absenteeism and travel time were common. A recent systematic review and meta-analysis also found that anti-VEGF therapy improved vision-related quality of life, although the absolute gain was modest [109,110].

Prospective IPC studies should therefore include patient-reported visual function, quality of life, treatment satisfaction, adherence, caregiver burden, and direct and indirect treatment costs alongside BCVA, anatomy, progression, and safety.

## 10. Emerging Therapies: Beyond VEGF Toward Disease Modification

The remarkable success of anti-VEGF therapy has transformed the management of DRD over the past two decades. Nevertheless, important unmet needs remain. A substantial proportion of patients demonstrate incomplete anatomical or functional responses, require lifelong intravitreal therapy, or continue to experience disease progression despite adequate VEGF suppression. These limitations have stimulated the development of novel therapeutic strategies aimed at targeting additional pathogenic pathways, extending treatment durability, and ultimately modifying the biological course of DRD rather than simply controlling its clinical manifestations.

One of the most important recent advances has been the transition from single-target therapy toward modulation of multiple biological pathways. Although VEGF remains a central mediator of vascular permeability and angiogenesis, Ang-2 also contributes significantly to endothelial dysfunction, vascular instability, inflammation, and increased vascular permeability. Simultaneous inhibition of VEGF-A and Ang-2 therefore represents a more comprehensive strategy for vascular stabilization. Faricimab, the first bispecific antibody targeting both pathways, has demonstrated visual outcomes comparable to conventional anti-VEGF therapy while allowing extended treatment intervals in many patients. This paradigm illustrates how modulation of complementary molecular pathways may improve treatment durability without compromising efficacy [7,21].

Improving treatment durability has become another major therapeutic objective. The cumulative burden associated with repeated intravitreal injections remains one of the greatest challenges in the long-term management of DRD. Accordingly, multiple sustained drug-delivery strategies are under active investigation, including biodegradable implants, refillable intraocular reservoirs, polymer-based delivery systems, hydrogel technologies, and gene therapy approaches designed to provide continuous intraocular production of therapeutic proteins. Although many of these technologies remain investigational, they have the potential to substantially reduce treatment burden while maintaining stable therapeutic drug concentrations within the retina [51,111].

Beyond vascular regulation, increasing attention has focused on therapies directed at chronic inflammation. Numerous inflammatory mediators—including interleukins, tumor necrosis factor-α, complement components, inflammasome signaling, and microglial activation pathways—contribute to retinal injury throughout disease progression. Future anti-inflammatory therapies may provide more selective modulation of these pathways than currently available corticosteroids while minimizing steroid-related adverse effects. Likewise, complement inhibition has emerged as a promising therapeutic strategy in light of growing evidence implicating innate immune dysregulation in DRD [13,18,112].

Neuroprotection represents another rapidly expanding area of investigation. Increasing recognition that retinal neurodegeneration may precede clinically apparent microvascular abnormalities has shifted attention toward therapies capable of preserving neuronal integrity before irreversible structural damage occurs. Experimental approaches—including neurotrophic factors, mitochondrial protection, modulation of oxidative stress, stem cell-based therapies, regenerative medicine, and cell-based replacement strategies—seek not only to prevent vision loss but also to preserve retinal function throughout disease progression. Although these therapies remain largely investigational, they emphasize that DRD is fundamentally a neurovascular disorder rather than a purely vascular disease [14,51,113].

Advances in molecular medicine are also paving the way toward truly personalized therapy. Multiomic technologies—including genomics, transcriptomics, proteomics, metabolomics, and AI-assisted biomarker discovery—are expected to identify individual molecular signatures associated with disease progression, treatment response, and prognosis. Integration of these data with multimodal retinal imaging and systemic clinical characteristics may eventually allow clinicians to select the most appropriate treatment before therapy is initiated, reducing empirical treatment strategies and improving long-term outcomes [114,115,116].

Ultimately, the future of DRD management will likely depend less on the discovery of a single superior drug than on the intelligent integration of complementary therapeutic strategies. Pharmacological therapies, sustained drug-delivery systems, systemic metabolic optimization, LP, vitreoretinal surgery, and emerging biological treatments should be regarded as complementary interventions directed toward distinct pathogenic mechanisms rather than competing alternatives. Within the framework of IPC, success will be measured not only by improvements in retinal thickness or visual acuity but also by the ability to achieve durable disease modification through individualized therapeutic selection, reduced treatment burden, and preservation of long-term retinal function.

Rather than replacing existing therapies, future innovations will expand the therapeutic toolbox available for individualized care. The challenge will therefore shift from identifying the best treatment to selecting the optimal combination of therapies for each patient at the appropriate stage of disease.

## 11. Artificial Intelligence and Future Biomarkers: The Next Frontier in Precision Care

### 11.1. Current and Near-Term Clinical Applications

AI is increasingly used in DRD, but maturity varies substantially by application. The most clinically mature uses involve automated screening and grading, lesion detection and segmentation, and quantitative retinal image analysis. These applications should be distinguished from therapeutic decision support, multiomic integration, foundation models, and digital twins, which remain translational, investigational, or conceptual.

The current and emerging applications of AI throughout the continuum of DRD management are summarized in Table 6. Beyond automated screening and diagnosis, AI is increasingly expected to integrate multimodal retinal imaging, systemic clinical information, and molecular biomarkers to support biological phenotyping, prognostication, and individualized therapeutic decision-making within the IPC framework [117,118,119,120].

The first major clinical success of AI in DRD has been population-based screening and grading using fundus photography. Contemporary systems can also support lesion detection, segmentation, and quantitative analysis of OCT and OCTA. Within IPC, currently mature AI should therefore be regarded primarily as a tool for detection, classification, quantitative image analysis, and workflow support rather than an autonomous system for selecting individualized ocular therapy [121].

Beyond diagnosis, AI is increasingly being applied to multimodal retinal imaging. Advanced algorithms can simultaneously analyze structural OCT, OCTA, ultra-WFI, and color fundus photography, identifying imaging biomarkers that may be difficult to detect through conventional clinical interpretation. Automated quantification of retinal thickness, intraretinal fluid, hyperreflective retinal foci, DRIL, photoreceptor integrity, retinal perfusion, ischemic burden, and vascular density has the potential to improve reproducibility, reduce observer variability, and facilitate objective phenotypic characterization. These capabilities may ultimately support more individualized therapeutic selection and disease monitoring [122,123,124].

### 11.2. Experimental and Future Precision Care Technologies

AI-guided therapeutic selection and prediction of individual pharmacologic response remain investigational. Although risk-prediction models may support surveillance research, prospective evidence demonstrating that AI improves treatment selection or clinical outcomes beyond conventional care is still required.

Multimodal foundation models that integrate imaging, electronic health records, laboratory data, and other modalities are experimental. Their potential to support individualized prediction is scientifically attractive, but external validation, calibration, interpretability, data governance, and demonstration of incremental clinical utility are prerequisites for routine use.

Patient-specific digital twins remain conceptual or early-stage research tools in ophthalmology. Their proposed ability to simulate disease trajectories or therapeutic scenarios should be regarded as a future research direction rather than an available clinical component of IPC.

Multiomic integration—including genomics, transcriptomics, proteomics, metabolomics, and epigenomics—also remains predominantly research-stage in DRD. Molecular signatures may eventually refine biological phenotyping, but standardized acquisition, external validation, cost, accessibility, and demonstration of treatment-predictive value are required before routine therapeutic use.

Despite these remarkable advances, several challenges must be addressed before AI can be fully integrated into routine clinical practice. Algorithm transparency, external validation, dataset diversity, regulatory oversight, data privacy, interoperability, and seamless incorporation into clinical workflows remain essential prerequisites for widespread adoption. Importantly, AI should be viewed as a clinical decision-support system rather than a replacement for physician expertise. Clinical judgment will remain indispensable for interpreting complex cases, incorporating patient preferences, and balancing therapeutic risks and benefits within the broader clinical context [122,125,126,127,128].

AI may eventually provide part of the technological infrastructure required for IPC; however, its current clinically mature role in DRD remains predominantly centered on screening, classification, and image analysis. Therapeutic decision support, multimodal biological integration, foundation models, and digital twins require prospective validation before they can be considered routine components of individualized retinal care.

This therapeutic evolution is also reflected across complementary clinical evidence spanning subthreshold laser and combination strategies for DME, recognition of the multifactorial nature of DME, systemic–retinal interactions measurable by OCT angiography, and more recent efforts to extend anti-VEGF durability with individualized brolucizumab dosing and aflibercept 8 mg [8,52,53,79,81,99]. Collectively, these studies reinforce the central premise of IPC: DRD is best approached as a heterogeneous, dynamic neurovascular disorder in which treatment selection and durability should be adapted to biological phenotype, systemic context, and patient-specific needs.

## 12. Conclusions and Future Perspectives: Integrated Precision Care as a New Therapeutic Paradigm

### 12.1. Why Current Treatment Algorithms Are No Longer Sufficient

The therapeutic landscape of DRD has undergone a profound transformation over the past two decades. The introduction of intravitreal anti-VEGF therapy dramatically improved visual outcomes and established a new standard of care for DME and PDR. Nevertheless, despite these remarkable advances, current treatment algorithms remain largely based on standardized protocols that assume patients with similar anatomical findings should receive similar therapeutic interventions.

Accumulating clinical experience and an expanding body of evidence demonstrate that DRD is biologically heterogeneous. Patients presenting with comparable retinal thickness, similar grades of DR, or equivalent visual acuity frequently exhibit markedly different rates of disease progression, treatment responses, therapeutic burden, and long-term visual outcomes. This variability reflects the complex interaction of multiple pathogenic mechanisms—including vascular dysfunction, chronic inflammation, retinal ischemia, neurodegeneration, vitreoretinal interface abnormalities, and systemic metabolic factors—that contribute differently in each individual patient.

Collectively, these observations challenge the traditional one-size-fits-all therapeutic paradigm and support the need for a more individualized strategy capable of addressing the biological complexity of DRD.

### 12.2. From Uniform Treatment to Integrated Precision Care

Based on the evidence reviewed throughout this article, we propose IPC as a conceptual and research framework—not a prospectively validated treatment algorithm—for DRD. Current treatment decisions should continue to follow established evidence-based indications for DME, NPDR, and PDR. IPC provides a structured way to generate and test hypotheses about whether imaging, systemic, pharmacologic, delivery, and patient-centered information can add value beyond conventional stage-based care.

Rather than treating DRD as biologically uniform, IPC recognizes overlapping candidate phenotypes while preserving conventional clinical staging. Phenotype–treatment relationships should be interpreted as testable hypotheses unless supported by established indications or demonstrated treatment-biomarker interactions.

Within this framework, conventional disease stage and the immediate vision-threatening indication take precedence. Imaging, systemic status, prior response, pharmacologic exposure profile, treatment burden, safety, access, and patient preference can then refine individualized management.

Consequently, multimodal retinal imaging extends beyond diagnosis to become a tool for biological phenotyping. Structural OCT, OCTA, ultra-WFI, and future imaging technologies provide biomarkers reflecting inflammation, vascular permeability, ischemia, neurodegeneration, and vitreoretinal interface abnormalities. Simultaneously, systemic factors—including glycemic control, hypertension, renal dysfunction, dyslipidemia, obesity, cardiovascular health, and anticipated treatment adherence—directly influence both disease progression and therapeutic response.

Within this paradigm, the central clinical question is no longer:

Which treatment is best for DRD?

Instead, clinicians should ask:

Which treatment is most appropriate for this patient’s biological phenotype at this specific stage of disease?

This conceptual transition shifts the research question from whether one protocol is universally optimal to whether multidimensional, evidence-weighted information can improve outcomes beyond conventional stage-based management.

### 12.3. Precision Phenotyping

For research purposes, candidate IPC phenotypes should be operationalized using reproducible structural, vascular, and clinical features rather than presumed molecular mechanisms alone. These features are not universally validated treatment-selection thresholds. Quantitative cutoffs for several biomarkers remain device-, acquisition-, and population-dependent, particularly for OCTA vascular parameters and HRF.

Rather than classifying patients solely according to the presence of DME or PDR, future management should increasingly classify disease according to the predominant pathogenic mechanisms driving retinal injury. Although these biological processes frequently overlap, identifying the dominant mechanism provides a rational framework for therapeutic selection.

These phenotypes are not intended to replace conventional clinical staging but rather to complement it by identifying the predominant biological mechanisms that may be amenable to targeted therapeutic intervention.

Illustrative phenotypes include:

- VEGF/leakage-dominant candidate phenotype: center-involving retinal thickening with intraretinal and/or subretinal fluid on OCT in clinically active DME; CRT should be reported quantitatively using device-specific segmentation rather than a universal IPC cutoff.
- Inflammatory-associated candidate phenotype: persistent or recurrent DME with features associated with inflammatory activity, including HRF and selected cystic patterns; no validated HRF-number threshold currently establishes an inflammatory phenotype or steroid superiority.
- Ischemic candidate phenotype: macular or peripheral capillary nonperfusion on OCTA, fluorescein angiography, or UWF angiography, including FAZ enlargement or reduced vessel density relative to device/protocol references; no universal FAZ or vessel-density cutoff mandates treatment.
- Tractional candidate phenotype: OCT evidence of epiretinal membrane, vitreomacular traction, posterior hyaloid abnormalities, tractional distortion, or tractional retinal detachment; surgical relevance depends on anatomy, symptoms, function, and progression rather than the presence of an interface abnormality alone.
- Neurodegenerative/poor-prognosis candidate phenotype: DRIL, ellipsoid-zone or external-limiting-membrane disruption, retinal thinning, and other structural markers of neuroretinal damage; these features are primarily prognostic and do not currently define a validated neuroprotective treatment pathway.

Mixed or overlapping phenotypes are expected rather than exceptional. Coexistence of multiple features should not automatically trigger simultaneous multimodal treatment. The hierarchy is as follows: immediate threat to vision, established evidence-based indication, dominant clinically actionable process, and reassessment of residual disease activity before additional intervention.

### 12.4. Personalized Therapeutic Selection

Within IPC, phenotype identification does not directly mandate therapy. Established conditions posing an immediate threat to vision—such as active proliferative neovascularization, macula-threatening traction, or vision-impairing center-involved DME—should first be managed according to evidence-based indications. When more than one established indication coexists, treatment should be prioritized according to urgency, risk of irreversible visual loss, safety, and feasibility of combined or sequential therapy. After the principal sight-threatening process has been addressed, residual disease activity should be reassessed before considering additional phenotype-associated mechanisms.

Figure 1 illustrates this proposed research framework. Candidate phenotypes and biomarkers are linked to phenotype-informed considerations that require prospective validation, while conventional stage-specific indications, continuous monitoring, systemic risk-factor control, patient-centered care, and dynamic reassessment apply across phenotypes. The figure should not be interpreted as a validated clinical treatment algorithm.

Table 7 summarizes the same distinction between evidence-supported clinical management and phenotype-informed considerations within IPC. Evidence status is presented qualitatively to distinguish established therapeutic evidence from emerging associations; it does not represent a formal systematic evidence-grading process.

Equally important, systemic optimization should be coordinated with the appropriate diabetes, primary-care, renal, and cardiovascular teams. Advanced imaging, AI, multiomics, and other emerging technologies should be incorporated only according to evidence, availability, actionability, and research maturity rather than assumed to be routine requirements of IPC.

Accordingly, the objective of IPC research is to determine whether multidimensional phenotyping can improve outcomes beyond conventional evidence-based care without increasing unnecessary treatment, cost, or inequity.

### 12.5. Operational Definition of Disease Modification

In this review, disease modification is defined as a durable alteration in the natural trajectory of DRD that reduces the risk or rate of clinically meaningful progression beyond transient suppression of retinal edema, vascular leakage, or neovascular activity. Disease control refers to suppression of current disease activity, whereas treatment durability refers to maintenance of control with less frequent intervention.

Neither should automatically be equated with disease modification.

Candidate endpoints for prospective demonstration of disease modification include sustained reduction in progression to vision-threatening DRD; time to PDR or center-involved DME; durable stabilization or improvement in retinopathy severity after protocol-defined treatment reduction or withdrawal; preservation of visual function and retinal structural integrity; prevention of recurrent DME or proliferative complications; and reduced cumulative rescue treatment without loss of disease control. Patient-reported outcomes and treatment burden should be complementary endpoints. Short-term changes in retinal thickness, fluid, neovascular activity, or DRSS during active therapy should not, in isolation, be interpreted as proof of disease modification.

Longitudinal OCT, OCTA, angiographic, neuroretinal, or molecular measures may provide exploratory mechanistic endpoints, but most have not been validated as surrogate endpoints for disease modification and should not replace clinically meaningful functional or disease-progression outcomes.

### 12.6. Future Perspectives

Future DRD research will increasingly examine the convergence of advanced retinal imaging, pharmacologic and delivery innovation, AI, molecular diagnostics, and patient-centered care. Their clinical value should be established incrementally rather than assumed from technological capability.

AI and multiomic technologies may eventually integrate imaging, systemic, longitudinal, and molecular information into predictive models. At present, however, therapeutic-response prediction and multiomic treatment selection remain investigational and require prospective validation before routine implementation.

Future therapeutic success will therefore depend less on the discovery of a single superior drug than on increasingly accurate patient selection.

Ultimately, IPC should be regarded as a structured research framework rather than another treatment algorithm. Its proposed value lies in organizing established and emerging evidence around the question of whether the right intervention, exposure profile, and follow-up strategy can be better matched to the right patient without displacing evidence-based stage-specific care.

As biomarkers, therapeutic platforms, formulations, and computational methods evolve, IPC can serve as a flexible structure for prospective testing. Its clinical utility, superiority, cost-effectiveness, and generalizability remain to be demonstrated.

Rather than representing a final clinical destination, IPC provides a hypothesis-generating platform for translational research in DRD. Prospective comparison with conventional evidence-based care is required before routine implementation can be recommended.

## 13. Limitations of the Review and the IPC Framework

This review has several limitations that should be considered when interpreting the proposed IPC framework. As a narrative rather than a systematic review, although landmark trials, systematic reviews, contemporary guidelines, and recent clinically relevant evidence were prioritized, study identification and selection were not based on a prospectively registered systematic-search protocol and may therefore be influenced by author judgment. The review should not be interpreted as an exhaustive or formally weighted synthesis of all available evidence.

The evidence integrated within IPC is heterogeneous, ranging from randomized clinical trials and meta-analyses to observational studies, exploratory biomarker investigations, and conceptual or preclinical work. Inclusion within one framework does not imply equivalent evidentiary weight. Many proposed imaging biomarker–treatment relationships remain associative rather than prospectively validated as predictive. Differences among devices, acquisition protocols, segmentation methods, biomarker definitions, and patient populations further limit universal quantitative thresholds.

Several potential future components of IPC—including multiomics, AI-guided therapeutic selection, foundation models, and digital twins—remain investigational or conceptual. Their incremental clinical value, accessibility, cost-effectiveness, regulatory requirements, and integration into routine workflows require prospective evaluation.

IPC itself has not been prospectively validated and should not be interpreted as a clinical practice guideline or replacement for established stage-specific management of DME, NPDR, or PDR. The phenotype definitions, prioritization rules, and proposed integration of ocular and systemic variables are hypothesis-generating. Prospective studies should compare IPC-informed approaches with conventional evidence-based care using predefined visual, anatomical, progression, safety, patient-reported, treatment-burden, equity, and health-economic outcomes across diverse populations and healthcare systems.

The principal clinical implications of the proposed IPC framework are summarized in Box 1.

Box 1 **Clinical Take-Home Messages.**

- DRD is a biologically heterogeneous neurovascular disorder rather than a purely VEGF-driven vascular disease.
- Multimodal retinal imaging supports diagnosis, prognosis, phenotypic characterization, and longitudinal monitoring; most biomarker-guided treatment-selection rules remain unvalidated.
- Anti-VEGF therapy remains the cornerstone of treatment for many patients but should not be regarded as the universal therapeutic solution.
- Corticosteroids, laser, vitreoretinal surgery, and systemic risk-factor optimization have established roles in selected clinical contexts; phenotype-guided extension beyond established indications requires validation.
- MIB provides objective information for prognosis and monitoring, but association with a biological mechanism does not by itself establish predictive treatment utility.
- Future precision-care research should test whether phenotype-informed strategies improve outcomes beyond conventional evidence-based stage-specific management.
- IPC is a proposed research framework integrating staging, imaging, systemic health, pharmacology and drug delivery, patient-specific factors, and emerging technologies; it is not a validated clinical algorithm.
- The ultimate investigational goal of IPC is to determine whether individualized therapeutic selection can achieve durable disease modification beyond conventional disease control.

## Figures and Tables

**Table 1:** Evolution of Therapeutic Strategies in Diabetic Retinal Disease.

Therapeutic Era	Primary Biological Target	Representative Treatment	Main Limitation	Current Role
Laser era	Retinal ischemia	PRP/focal-grid laser	Limited visual improvement	Selected indications
Anti-VEGF era	VEGF	Ranibizumab, aflibercept, bevacizumab	Frequent intravitreal injections	Standard first-line therapy
Dual-pathway inhibition	VEGF + Ang-2	Faricimab	Incomplete response in some patients	Extended durability
Sustained drug delivery	Continuous intraocular therapy	Corticosteroid implants, refillable delivery systems	Device-related limitations	Selected patients
Disease-modifying therapies	Multiple pathogenic pathways	Gene therapy, neuroprotection, regenerative medicine	Under investigation	Future perspective

Abbreviations. Ang-2 = angiopoietin-2; PRP = panretinal photocoagulation; VEGF = vascular endothelial growth factor.

**Table 2:** Multimodal Imaging Biomarkers: Current Role, Therapeutic Implication, and Evidence Status.

Imaging Biomarker	Clinical/Biological Association	Current Biomarker Role	Therapeutic Implication	Evidence Status
Increased CRT/IRF/SRF	Active DME/exudative activity	Disease activity and monitoring	Supports assessment of response; does not independently select a specific drug	Established for monitoring; agent-specific prediction limited
HRF	Associated with inflammatory activity and worse visual prognosis	Predominantly prognostic/phenotypic	Steroid-selection role remains emerging; HRF alone should not dictate therapy	Emerging/unvalidated predictive role
DRIL	Neuroretinal disorganization	Prognostic	No validated treatment-selection role	Moderate prognostic evidence; predictive role limited
EZ/ELM disruption	Photoreceptor/outer-retinal injury	Prognostic	No validated treatment-selection role; informs counseling	Moderate prognostic evidence
Large intraretinal cysts	Chronic edema/structural injury	Disease characterization/prognosis	May inform assessment of chronicity; no validated drug-specific rule	Emerging
FAZ enlargement/reduced vessel density	Macular ischemia/capillary loss	Prognosis and risk stratification	No universal ischemia-directed treatment threshold	Emerging; device-dependent
Peripheral nonperfusion	Peripheral ischemic burden	Progression-risk marker	Surveillance; treat only when an established stage-specific indication exists	Prognostic; treatment-predictive role limited
Retinal neovascularization	Active proliferative disease	Clinical disease activity	PRP and/or anti-VEGF according to established PDR indications	High clinical evidence
ERM/VMT/traction	Mechanical vitreoretinal-interface abnormality	Anatomic/surgical assessment	Contributes to surgery when clinically significant; interface abnormality alone is insufficient	Established for selected surgical indications

Abbreviations. CRT = central retinal thickness; DME = diabetic macular edema; HRF = hyperreflective retinal foci; DRIL = disorganization of the retinal inner layers; EZ = ellipsoid zone; ELM = external limiting membrane; SRF = subretinal fluid; ERM = epiretinal membrane; VMT = vitreomacular traction; FAZ = foveal avascular zone; OCTA = optical coherence tomography angiography; UWF = ultra-widefield imaging; PRP = panretinal photocoagulation.

**Table 3:** Selected quantitative evidence from pivotal and contemporary DME trials, including sample size, regimen and follow-up, visual and anatomic outcomes, treatment burden or durability, and key safety information.

Trial	N	Regimen/Follow-Up	Visual Outcome	Anatomic Outcome	Treatment Burden/Durability	Key Safety Note
RISE/RIDE	377/382	Ranibizumab monthly; 24 mo	≥15-letter gain: 33.6–44.8% with ranibizumab 0.3 mg vs. 12.3–18.1% sham	Significant OCT improvement	Monthly treatment	Endophthalmitis occurred in 4 ranibizumab-treated patients across trials
Protocol T	660	Aflibercept/bevacizumab/ranibizumab; 1 y	+13.3/+9.7/+11.2 letters; in baseline VA ≤ 20/50: +18.9/+11.8/+14.2	CST reduction favored aflibercept over bevacizumab in key subgroups	~9–10 injections in year 1	Comparable ocular/systemic safety across agents
YOSEMITE/RHINE	1891	Faricimab Q8W or personalized up to Q16W vs. aflibercept Q8W; 1–2 y	Noninferior BCVA gains; pooled year-2 gains ~+10 letters	Greater/faster anatomic control with faricimab in pooled analyses	78.1% of pooled faricimab T&E cohort on ≥Q12W at week 96	Ocular AE incidence broadly comparable to aflibercept
KESTREL/KITE	566/360	Brolucizumab 6 mg vs. aflibercept; 100 wk	+8.8 vs. +10.6 letters (KESTREL); +10.9 vs. +8.4 (KITE)	Robust fluid control	32.9% KESTREL on Q12W; 47.5% KITE on Q12W/Q16W	Drug-specific IOI/retinal vasculitis/vascular occlusion signal
PHOTON	658	Aflibercept 8 mg Q12/Q16 vs. 2 mg Q8; 96 wk	+8.8/+7.5 vs. +8.4 letters	CRT −185/−155 vs. −187 micrometers	9.5/7.8 vs. 13.8 injections; many maintained ≥ 12–16 wk	Ocular treatment-emergent AEs similar across groups
PAGODA	634	PDS ranibizumab Q24W vs. monthly ranibizumab; 64 wk	+9.6 vs. +9.4 letters	Maintained disease control	Refill-exchange every 24 wk	Device/procedure AESIs more common with PDS; no endophthalmitis or RD reported

**Table 5:** Systemic and Patient-Specific Factors Supporting Resource-Adapted Integrated Precision Care.

Systemic/Patient Factor	Relevance to DRD	Representative Target or Management Principle	IPC Implication
Glycemic control	DR development/progression	HbA1c < 7% for many nonpregnant adults; individualized	Primary care/endocrinology; not a retina-specific threshold
Hypertension	DR progression/vascular risk	<130/80 mmHg when safely achievable in diabetes with hypertension; individualized	Primary care/cardiology/nephrology as appropriate
Dyslipidemia	Hard exudates/DR risk	Risk-based lipid management; no single retina-specific LDL threshold	Coordinate cardiovascular-risk management; fenofibrate only in selected contexts
Chronic kidney disease/albuminuria	Associated with more advanced DRD and systemic risk	Assess eGFR and urinary albumin according to diabetes/CKD guidance	Nephrology involvement when clinically indicated
Obesity/lifestyle	Cardiometabolic and inflammatory risk	Individualized weight, nutrition, physical activity, sleep, smoking cessation	General diabetes/cardiovascular care; no retina-specific cutoff
Treatment adherence/access	Undertreatment and poorer real-world outcomes	Assess travel, work, caregiver, financial and visit burden	Consider durable regimens when efficacy/safety support them
Lens/glaucoma status	Modifies corticosteroid risk-benefit	Assess pseudophakia, IOP, glaucoma and prior steroid response	Ocular selection variable, not systemic target

Abbreviations. DR = diabetic retinopathy; HbA1c = glycated hemoglobin.

**Table 6:** Current maturity and potential future role of artificial intelligence and related technologies in diabetic retinal disease. Screening, grading, and image analysis are more clinically mature than treatment-selection AI, multiomics integration, foundation models, and digital twins.

Technology/Application	Current Maturity	Present Role	Potential IPC Role
Automated fundus screening/grading	Clinical/validated	Detection and referral	Scalable case identification
Lesion detection/segmentation	Clinical to translational	Quantitative image analysis	Standardized phenotyping support
OCT/OCTA AI analysis	Translational	Biomarker quantification/research	Multimodal phenotyping research
Risk prediction	Translational	Selected prognostic models	Individualized surveillance research
AI-guided treatment selection	Investigational	Research only	Therapy-response prediction requires prospective validation
Multiomics + AI	Experimental	Research	Molecular phenotyping
Foundation models	Experimental	Research	Multimodal integration; external validation required
Digital twins	Conceptual/experimental	No established routine role	Patient-specific simulation research

**Table 7:** Proposed research framework for phenotype-informed management of diabetic retinal disease. Evidence status is qualitative and distinguishes established therapeutic evidence from emerging phenotype-treatment associations; it does not represent a formal systematic evidence-grading process. The table is not a validated clinical algorithm or practice guideline, and established stage-specific treatment indications remain governed by current evidence-based recommendations.

Clinical Phenotype	Candidate Imaging/Clinical Features	Evidence-Supported Clinical Management	Phenotype-Informed Consideration Within IPC	Evidence Status
VEGF-dominant DME	Center-involving edema; IRF/SRF; increased CRT	Anti-VEGF for vision-impairing center-involved DME	Individualize agent/dosing by VA, response, durability, burden, and safety	High for anti-VEGF; emerging for biomarker-guided agent selection
Inflammatory-associated DME	Persistent/recurrent edema; HRF or selected cystic patterns may be associated with inflammation	Anti-VEGF remains standard initial therapy in most eyes; corticosteroids established in selected patients	Inflammatory features may support research stratification together with lens status, IOP/glaucoma risk and prior response	Moderate-high for steroid efficacy in selected DME; emerging for imaging-guided selection
Ischemic/neovascular	Nonperfusion, FAZ enlargement, reduced vessel density; neovascularization when present	Treat DME, PDR/neovascularization, or selected high-risk severe NPDR according to established indications	Use ischemic burden mainly for prognosis, risk stratification, and monitoring; ischemia alone does not routinely mandate PRP/anti-VEGF	High for established PDR treatment; limited for isolated ischemia-directed intervention
Tractional/VMI	ERM, VMT, posterior hyaloid abnormalities, tractional distortion/detachment	Vitrectomy for established surgical indications	Earlier phenotype-guided surgery outside conventional indications remains a research hypothesis	High for established indications; limited for earlier phenotype-guided surgery
Neurodegenerative/poor prognosis	DRIL, EZ/ELM disruption, retinal thinning	Optimize established stage-specific DR/DME therapy and systemic factors	Neuroprotective treatment selection based on imaging biomarkers remains investigational	Prognostic evidence present; predictive/therapeutic evidence limited
Mixed/overlapping	Features from ≥2 domains	Treat the established indication posing the greatest immediate threat to vision	Reassess residual disease activity and consider sequential management only for additional clinically actionable mechanisms	Variable; IPC prioritization conceptual and requires prospective validation

## Data Availability

No new data were created or analyzed in this study. Data sharing is not applicable to this article.
